# Serum neurofilament light chain levels in patients with cognitive deficits and movement disorders: comparison of cerebrospinal and serum neurofilament light chain levels with other biomarkers

**DOI:** 10.3389/fnhum.2023.1284416

**Published:** 2023-12-14

**Authors:** Richard Novobilský, Petra Bartova, Karin Lichá, Michal Bar, David Stejskal, Pavlína Kusnierova

**Affiliations:** ^1^Department of Neurology, University Hospital Ostrava, Ostrava, Czechia; ^2^Department of Clinical Neurosciences, University of Ostrava, Ostrava, Czechia; ^3^Department of Clinical Biochemistry, Institute of Laboratory Medicine, University Hospital Ostrava, Ostrava, Czechia; ^4^Institute of Laboratory Medicine, University of Ostrava, Ostrava, Czechia

**Keywords:** neurofilament light chain, high-sensitivity ELISA, cognitive deficit, mini-mental state examination, serum

## Abstract

**Background:**

Serum neurofilament light chain (S NfL) is a non-specific marker of neuronal damage, including Alzheimer’s disease (AD). We aimed to verify the reference interval (RI) of serum NfL using a highly sensitive ELISA, and to estimate the optimal cut-off value for neuronal damage. Our second objective was to compare NfL in cerebrospinal fluid (CSF) and serum (S) with the routine neurodegeneration biomarkers used in AD, and to assess their concentrations relative to the degree of cognitive deficit.

**Methods:**

Samples from 124 healthy volunteers were used to estimate the S NfL RI. For the comparison study, we used CSF and S samples from 112 patients with cognitive disorders. Cognitive functions were assessed using the mini-mental state examination. ELISA assays were used to determine the CSF and S NfL levels, CSF β-amyloid peptide_42_ (Aβ_42_), CSF β-amyloid peptide_40_ (Aβ_40_), CSF total tau protein (tTau), CSF phosphorylated tau protein (pTau), and CSF alpha-synuclein (αS).

**Results:**

The estimated RI of S NfL were 2.25–9.19 ng.L^–1^. The cut-off value of S NfL for assessing the degree of neuronal impairment was 10.5 ng.L^–1^. We found a moderate statistically significant correlation between S NfL and CSF Aβ_42_ in the group with movement disorders, without dementia (*r*_*s*_ = 0.631; *p* = 0.016); between S NfL and CSF Aβ_40_ in the group with movement disorder plus dementia (*r*_*s*_ = −0.750; *p* = 0.052); between S NfL and CSF tTau in the control group (*r*_*s*_ = 0.689; *p* = 0.009); and between S NfL and CSF pTau in the control group (*r*_*s*_ = 0.749; *p* = 0.003). The non-parametric Kruskal–Wallis test revealed statistically significant differences between S NfL, CSF NfL, CSF Aβ_42_, CSF tTau, and CSF pTau and diagnosis within groups. The highest kappa coefficients were found between the concentrations of S NfL and CSF NfL (κ = 0.480) and between CSF NfL and CSF tTau (κ = 0.351).

**Conclusion:**

Our results suggested that NfL and tTau in CSF of patients with cognitive decline could be replaced by the less-invasive determination of S NfL using a highly sensitive ELISA method. S NfL reflected the severity of cognitive deficits assessed by mini-mental state examination (MMSE). However, S NfL is not specific to AD and does not appear to be a suitable biomarker for early diagnosis of AD.

## 1 Introduction

Neurodegenerative diseases constitute a broad group of diseases of the nervous system. They typically involve the progressive and irreversible loss of specific neuron populations in distinct brain and spine localizations, which result in diverse clinical manifestations, most often dementia and movement disorders. It has recently been theorized that the neurodegeneration process is caused by the precipitation of specific proteins in the tissue, leading to inflammation and apoptosis (Dugger and Dickson, 2017; Ciccocioppo et al., 2020).

Neurodegenerative diseases are most common in people over 65 years of age, their prevalence increases with age, and it is estimated that the number of people with dementia will double in the next 30 years due to increasing life expectancy (Ferri et al., 2005; Nichols et al., 2019). Therefore, laboratory biomarkers are being sought to help diagnose neurodegenerative disease in its early stages. In addition to the determination of β-amyloid_42_ (Aβ_42_), total tau protein (tTau), and phosphorylated tau protein_181_ (pTau), attention is now focused on evaluation of neurofilaments (Nf). The major structural proteins of neurons, Nfs are class IV intermediate filaments that are selectively expressed in neurons. Abnormal concentrations have been observed with axonal damage in neurodegenerative, inflammatory, vascular, and traumatic diseases, in both cerebrospinal fluid (CSF) and serum (S). Due to their high specificity for neuronal cell damage and eventual death, Nf levels are an important parameter for monitoring and predicting the progression of various acute and chronic neurological diseases, and for evaluating the efficacy of therapy (Khalil et al., 2018; Revendova et al., 2022).

The main objective of the present study was to verify the reference interval (RI) of serum neurofilament light chain (NfL) in a control population, using a highly sensitive serum ELISA, and to estimate the optimal cut-off value indicating neuronal damage. The second objective was to compare the determination of NfL in CSF and S, using a highly sensitive ELISA method, with the routine neurodegeneration biomarkers measured in Alzheimer’s disease (AD), and to study their concentrations relative to the degree of cognitive deficit among patients with neurodegenerative diseases.

## 2 Materials and methods

### 2.1 Patients and data collection

To determine the reference values for S NfL, serum samples were collected from healthy adults from the Blood Centre of the University Hospital Ostrava (*n* = 124, average age 43.8 ± 10.1 years; 60 females, average age 43.4 ± 10.0 years; 64 males, average age 44.1 ± 10.4 years). Outlying results (*n* = 8) were excluded from further statistical processing of the data. Except for gender and age, all other patient data were anonymous. All volunteers were healthy and were not taking any medication.

For comparative analysis, CSF and S samples were collected from patients attending the Outpatient Clinic for Movement and Cognitive Disorders at the University Hospital Ostrava, Czechia, who were included in a single-center prospective cohort study (*n* = 115, average age 67.1 ± 11.5 years; 66 females, average age 67.7 ± 11.5 years; 49 males, average age 66.2 ± 11.5 years). CSF of standardized volume (10 ± 1 ml) from all patients was obtained through a lumbar puncture in intervertebral space L3/L4, L4/5 or L5/S1. The inclusion criteria were: (1) gave signed informed consent for study inclusion; (2) brain imaging (CT or MRI) performed to exclude space-occupying brain lesions (e.g., tumor, brain contusion, multiple sclerosis, normal-pressure hydrocephalus, and large postischemic or posthemorrhagic lesion); (3) other causes of cognitive deficit excluded by laboratory examination (e.g., ion imbalance, anemia, B12 hypovitaminosis, Wilson’s disease, and thyroid disorder); and (4) a mini-mental state examination (MMSE) score of ≤25/30 points, with a temporal aspect of at least 6 months of clinical symptoms affecting daily activities; or (5) a neurodegenerative movement disorder, with primary complaints other than dementia [Parkinson’s disease (PD), multiple system atrophy, progressive supranuclear palsy, etc.] without cognitive deficit (MMSE score of >25/30 points). The exclusion criterion was: age of <18 years. The baseline data of the prospective study were used for the study of cognitive deficit. The control group included patients with an MMSE score of >28/30 and without clinical signs of parkinsonism (tremor, rigidity, bradykinesia, hypokinesia, and gait disturbance) following examination by an experienced neurologist. The cut-off score of 25 points on the MMSE was established according to the limitation of reimbursement by Czech insurance for treatment of AD. Five patients with severe dementia could not complete the MMSE because of their non-cooperation.

Patients were subdivided into groups: Group 1, AD according to NIA-AA research criteria for AD (Jack et al., 2018) (*n* = 33; average age 71.3 ± 9.2 years); Group 2, non-Alzheimer’s dementia (*n* = 32; average age 70.4 ± 9.9 years); Group 3, PD and patients with movement disorder without cognitive deficit (*n* = 24; average age 62.8 ± 10.9 years); Group 4, combination of cognitive syndrome and movement disorder (*n* = 10; average age 67.8 ± 13.1 years); and Group 5, healthy controls (*n* = 16; average age 57.6 ± 11.7 years). The diagnoses in Group 1 included AD according to NIA-AA research criteria for AD without dementia (*n* = 5), AD according to NIA-AA research criteria for AD (*n* = 9), and AD established by an experienced neurologist when patients did not agree to undergo lumbar puncture (*n* = 19). In Group 2, the Non-Alzheimer dementias comprised vascular dementia (*n* = 15); frontotemporal dementia (FTD, *n* = 8), Lyme neuroborreliosis (*n* = 3), alcohol-related dementia (*n* = 3), Creutzfeldt-Jakob disease (CJD, *n* = 2), and primary progressive aphasia (*n* = 1). Group 3 comprised patients with PD established by an experienced neurologist according to the criteria of the Movement Disorder Society (Postuma et al., 2015) (*n* = 14) and patients with movement disorders other than PD without dementia (*n* = 10) including multiple system atrophy (*n* = 4), progressive supranuclear palsy (*n* = 2), dystonia (*n* = 2), Huntington’s disease (*n* = 1), and essential tremor plus syndrome (*n* = 1). Group 4 Combination of cognitive syndrome and movement disorder consisted of patients with Lewy body disease (*n* = 6), Multiple system atrophy (*n* = 2), Spinocerebellar ataxia (*n* = 1), and progressive supranuclear palsy (*n* = 1).

### 2.2 Samples

All CSF samples were collected with an atraumatic needle into polypropylene tubes (Sarstedt, Nümbrecht, Germany). Serum samples were collected into a Serum Gel with Clotting Activator tube (Sarstedt). CSF samples were centrifuged at 390 × *g* for 10 min at room temperature, and serum samples were centrifuged at 2,500 × *g* for 6 min at 4°C. Both the CSF and serum samples were aliquoted into at least three vials (0.3 ml per vial) and stored at −70°C until analysis.

### 2.3 Analytical methods

The concentrations of CSF NfL and S NfL were determined by ELISA assays (NF-light^®^ ELISA CE, REF. 10-7001; NF-light™ Serum ELISA RUO, REF. 20-8002, UmanDiagnostics, A Quanterix Company). The manufacturers stated that the limits of detection were 33 ng.L for CSF NfL, and 0.4 ng.L for S NfL. All samples were analyzed in duplicates. CSF NfL and S NfL were measured in 2× diluted CSF and 4× diluted serum.

Concentrations of other biomarkers of neurodegenerative damage were determined by ELISA methods using the following diagnostic kits: Total-Tau-ELISA, REF. EQ 6531-9601-L; Beta-Amyloid (1-42)-ELISA, REF. EQ 6521-9601-L; Beta-Amyloid (1-40)-ELISA, REF. EQ 6511-9601-L and pTau(181) ELISA, REF EQ-6591-9601-L (Euroimmun); and Alpha-Synuclein ELISA, REF EQ 6545-9601-L. Undiluted CSF samples were used. The detection limits were 28 ng.L^–1^ for CSF tTau, 1.5 ng.L^–1^ for CSF pTau, 41 ng.L^–1^ for CSF Aβ_40_, 6.5 ng.L^–1^ for Aβ_42_, and 19 ng.L^–1^ for CSF αS.

### 2.4 Statistical methods

Microsoft Excel and MedCal version 17.9.7. were used for statistical data processing. Reference intervals (RI) were estimated based on the guidelines of the Clinical and Laboratory Standards Institute (CLSI C28-A3) (Clinical and Laboratory Standards Institute [CLSI], 2008; Pavlov et al., 2010). A robust method was used due to the sample size (*n* < 120).

The diagnostic value of S NfL was evaluated by receiver operating characteristics (ROC) curve analysis. The sensitivity and specificity, and 95% confidence interval (CI) were considered against the group involving any cognitive deficit. An area under the curve (AUC) of >0.9 was considered to have excellent diagnostic power.

Basic descriptive statistics were used to describe patient data, including tables of frequencies, minimum and maximum values, medians, arithmetic means, and standard deviations. Relationships between the biomarkers were assessed using Spearman’s correlation coefficient. The Shapiro–Wilk test was used to assess the normality of residues in the analysis of variance (ANOVA). Due to the non-normal distribution of data, non-parametric tests were used, including the Kruskal–Wallis rank test. The Dunn test was used for the *post hoc* analysis. The kappa statistic was used to assess the agreement between methods based on clinical interpretation (McHugh, 2012). Data values were categorized as positive and negative. Stepwise multinomial linear regression analysis was performed to assess the effects of biochemical markers on MMSE values. All statistical tests were assessed at the 5% significance level.

## 3 Results

### 3.1 Estimation of reference intervals, sensitivity, and specificity of S NfL using a highly sensitive ELISA method

**FIGURE 1:**
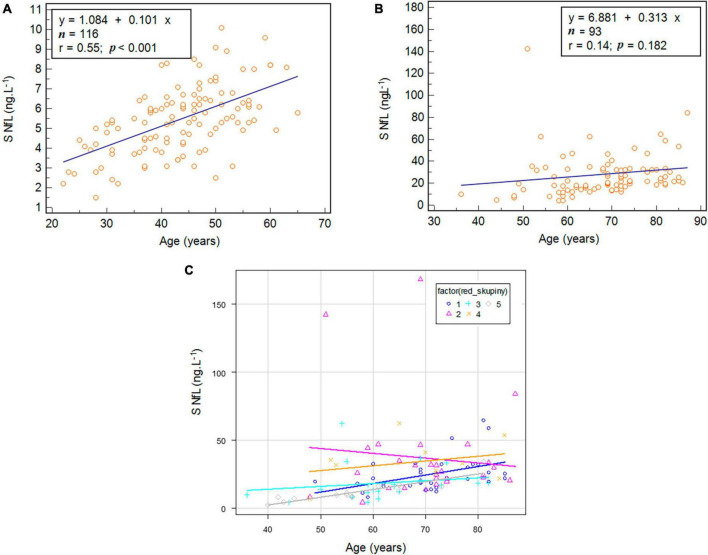
The age dependence of serum (S) neurofilament light chain (NfL) in the control group **(A)**, in patients from the Outpatient Clinic for Movement and Cognitive Disorders **(B)**, and in each diagnostic group **(C)**.

The total reference values of S NfL were 2.25–9.19 ng.L^–1^, and the data showed statistically significant age dependence (*r* = 0.55; *p* < 0.001) (Figure 1A). On the other hand, age dependence of S NfL was not observed among patients from the Outpatient Clinic for Movement and Cognitive Disorders (Figure 1B). A more detailed analysis revealed that the patients with a cognitive syndrome plus movement disorder (Group 4) exhibited a negative slope of the dependence of S NfL on age, which affected the outcome of the whole study population (Figure 1C).

**FIGURE 2:**
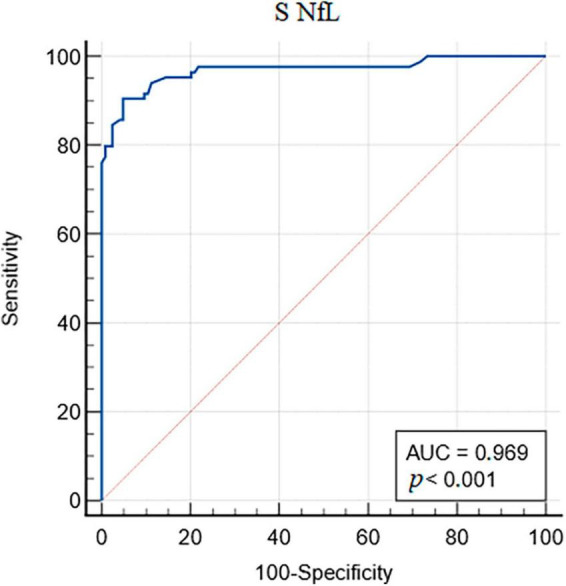
Receiver operating characteristic (ROC) analysis of serum (S) neurofilament light chain (NfL).

Receiver operating characteristics analyses revealed the optimal cut-off value of S NfL (>10.5 ng.L^–1^) for assessing the neuronal damage. The sensitivity was 90.5% (CI 82.1–95.8%), and specificity was 95.2% (CI 89.8–98.2%) (Figure 2).

### 3.2 Comparison of S NfL determined by highly sensitive ELISA with other biochemical markers in cohorts with different degrees of cognitive deficit

Samples from 115 patients were used for the comparison between methods. Table 1 presents characteristics of the studied group. The median concentration of S NfL was 20.1 ng.L^–1^ (IQR 14.0–32.3), CSF NfL 1036 ng.L^–1^ (IQR 639–2,363), CSF Aβ_40_ 7,779 ng.L^–1^ (IQR 5,376–9,964), CSF Aβ_42_ 1,127 ng.L^–1^ (IQR 661–1,562), CSF tTau 309 ng.L^–1^ (IQR 216–518), CSF pTau 41.2 ng.L^–1^ (IQR 27.7–71.3), and CSF αS 2,251 ng.L^–1^ (IQR 1,871–2,836).

**TABLE 1:** Descriptive characteristics of the studied groups.

Variable		*n*	Min	Max	Mean	Median	SD	*p*
Age (years)	F	66	40.0	86.0	67.7	69.0	11.5	0.051
	M	49	36.0	88.0	66.2	68.0	11.5	0.699
CSF NfL (ng.L^–1^)		76	239	10865	1856	1037	2071	<0.001
S NfL (ng.L^–1^)		105	2.30	168	26.3	20.1	23.3	<0.001
CSF Aβ_42_ (ng.L^–1^)		76	124	2502	1159	1127	533	0,165
CSF Aβ_40_ (ng.L^–1^)		60	357	15288	7829	7779	3189	0.657
CSF Aβ_42_/Aβ_40_		58	<0.01	3.73	0.22	0.18	0.47	<0.001
CSF tTau (ng.L^–1^)		76	86.1	2748	473	310	492	<0.001
CSF pTau (ng.L^–1^)		76	1.50	376	73.4	41.2	81.4	<0.001
CSF αS (ng.L^–1^)		59	19.0	28802	2836	2253	3583	<0.001
MMSE		110	9.00	30.0	23.2	24.0	5.94	<0.001

CSF NfL, cerebrospinal fluid neurofilament light chain; S NfL, serum neurofilament light chain; CSF Aβ_42_, cerebrospinal fluid β-amyloid peptide_42_; CSF Aβ_40_, cerebrospinal fluid β-amyloid peptide_40_; CSF tTau, cerebrospinal fluid total tau protein; CSF pTau, cerebrospinal fluid phosphorylated tau protein; αS, alpha-synuclein; MMSE, mini-mental state examination; *n*, number of patients; Min, minimal concentration; Max, maximal concentration; SD, standard deviation; *p*, test for normal distribution.

In all groups, we found a very strong Spearman’s rank correlation coefficient between the CSF NfL and S NfL (*r*_*s*_ = 0.767; *p* < 0.001) (Table 2). We found a moderate statistically significant correlation between S NfL and CSF Aβ_42_ in Group 3 (*r*_*s*_ = 0.631; *p* = 0.016); between S NfL and CSF Aβ_40_ in Group 4 (*r*_*s*_ = −0.750; *p* = 0.052); between S NfL and CSF tTau in Group 5 (*r*_*s*_ = 0.689; *p* = 0.009), and between S NfL and CSF pTau in Group 5 (*r*_*s*_ = 0.749; *p* = 0.003). Very strong correlation coefficients were found between CSF NfL and CSF Aβ_40_ in Group 4 (*r*_*s*_ = −0.964; *p* < 0.001); between CSF Aβ_42_ and CSF αS in Group 5 (*r*_*s*_ = 0.804; *p* = 0.002); between CSF Aβ_40_ and CSF tTau in Group 1 (*r*_*s*_ = 0.811 *p* = 0.001); between CSF Aβ_40_ and αS all groups (*r*_*s*_ = 0.811) in Group 1 (*r*_*s*_ = 0.909) and Group 5 (*r*_*s*_ = 0.868) (all *p* < 0.001); and between CSF tTau and αS in Group 1 (*r*_*s*_ = 0.937, *p* < 0.001).

**TABLE 2:** Correlations between selected biochemical markers in cerebrospinal fluid and serum in individual diagnostic groups.

		Diagnostic group					
		**All**	**(1)**	**(2)**	**(3)**	**(4)**	**(5)**
S NfL vs. CSF NfL	*r*_*s*_	0.767	0.341	0.619	0.835	0.881	0.890
	*p*	<0.001	0.213	0.002	<0.001	0.004	<0.001
	*n*	72	15	22	13	8	14
S NfL vs. CSF Aβ_42_	*r*_*s*_	−0.027	−0.129	0.254	0.631	−0.071	0.229
	*p*	0.822	0.610	0.255	0.017	0.879	0.452
	*n*	74	18	22	14	7	13
S NfL vs. CSF Aβ_40_	*r*_*s*_	−0.067	−0.466	−0.127	0.370	−0.750	0.077
	*p*	0.611	0.127	0.626	0.293	0.052	0.793
	*n*	60	12	17	10	7	14
S NfL vs. CSF tTau	*r*_*s*_	0.241	−0.025	−0.010	0.468	0.167	0.689
	*p*	0.039	0.922	0.967	0.091	0.693	0.009
	*n*	74	18	21	14	8	13
S NfL vs. CSF pTau	*r*_*s*_	0.180	−0.103	0.021	0.359	0.310	0.749
	*p*	0.125	0.694	0.927	0.208	0.456	0.003
	*n*	74	17	22	14	8	13
S NfL vs. CSF αS	*r*_*s*_	0.192	−0.343	0.348	0.273	−0.500	0.382
	*p*	0.146	0.275	0.171	0.446	0.253	0.197
	*n*	59	12	17	10	7	13
CSF NfL vs. CSF tTau	*r*_*s*_	0.401	0.589	0.323	0.462	0.071	0.754
	*p*	<0.001	0.021	0.154	0.112	0.867	0.002
	*n*	71	15	21	13	8	14
CSF NfL vs. CSF pTau	*r*_*s*_	0.161	0.396	−0.062	0.380	0.190	0.604
	*p*	0.178	0.144	0.786	0.201	0.651	0.022
	*n*	72	15	22	13	8	14
CSF NfL vs. CSF Aβ_42_	*r*_*s*_	0.036	−0.104	0.289	0.538	−0.357	0.327
	*p*	0.764	0.713	0.193	0.058	0.432	0.253
	*n*	71	15	22	13	7	14
CSF NfL vs. CSF Aβ_40_	*r*_*s*_	0.045	0.161	−0.079	0.515	−0.964	0.204
	*p*	0.736	0.618	0.770	0.128	<0.001	0.483
	*n*	59	12	16	10	7	14
CSF NfL vs. CSF αS	*r*_*s*_	0.374	0.329	0.438	0.685	−0.786	0.401
	*p*	0.004	0.297	0.090	0.029	0.036	0.174
	*n*	58	12	16	10	7	13
CSF Aβ_42_ vs. CSF tTau	*r*_*s*_	−0.017	0.326	0.391	0.560	0.429	0.644
	*p*	0.884	0.186	0.072	0.037	0.337	0.013
	*n*	75	18	22	14	7	14
CSF Aβ_42_ vs. CSF pTau	*r*_*s*_	−0.370	0.141	−0.309	0.224	0.464	0.459
	*p*	0.001	0.589	0.151	0.441	0.294	0.099
	*n*	75	17	23	14	7	14
CSF Aβ_42_ vs. CSF αS	*r*_*s*_	0.247	0.259	0.594	0.467	0.771	0.804
	*p*	0.066	0.417	0.015	0.174	0.072	0.002
	*n*	56	12	16	10	6	12
CSF Aβ_40_ vs. CSF tTau	*r*_*s*_	0.648	0.811	0.621	0.418	0.214	0.555
	*p*	<0.001	0.001	0.013	0.229	0.645	0.049
	*n*	57	12	15	10	7	13
CSF Aβ_40_ vs. CSF pTau	*r*_*s*_	0.444	0.315	0.335	0.370	0.107	0.302
	*p*	<0.001	0.319	0.204	0.293	0.819	0.316
	*n*	58	12	16	10	7	13
CSF Aβ_40_ vs. CSF αS	*r*_*s*_	0.811	0.909	0.620	0.758	0.750	0.868
	*p*	<0.001	<0.001	0.008	0.011	0.052	<0.001
	*n*	59	12	17	10	7	13
CSF tTau vs. CSF αS	*r*_*s*_	0.772	0.937	0.600	0.648	0.464	0.741
	*p*	<0.001	<0.001	0.018	0.043	0.294	0.006
	*n*	56	12	15	10	7	12
CSF pTau vs. CSF αS	*r*_*s*_	0.565	0.566	0.382	0.600	0.571	0.559
	*p*	<0.001	0.055	0.144	0.067	0.180	0.059
	*n*	57	12	16	10	7	12

CSF NfL, cerebrospinal fluid neurofilament light chain; S NfL, serum neurofilament light chain; CSF Aβ_42_, cerebrospinal fluid β-amyloid peptide_42_; CSF Aβ_40_, cerebrospinal fluid β-amyloid peptide_40_; CSF tTau, cerebrospinal fluid total tau protein; CSF pTau, cerebrospinal fluid phosphorylated tau protein; αS, alpha-synuclein.

### 3.3 Evaluation of the relationships between individual analytes and diagnosis

**FIGURE 3:**
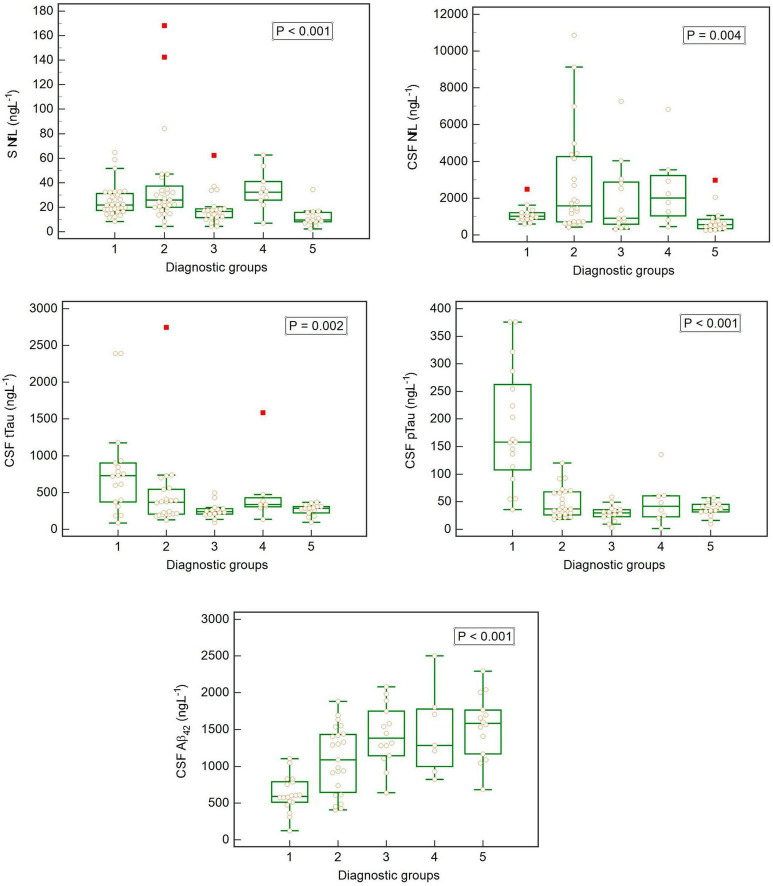
Box-plots showing concentrations of biomarkers in cerebrospinal fluid (CSF) and serum (S) in individual diagnostic groups (Kruskal–Wallis rank test).

The non-parametric Kruskal–Wallis test was used to evaluate the relationship between individual analytes and diagnosis. We found statistically significant differences in S NfL, CSF NfL, CSF Aβ_42_, CSF tTau, and CSF pTau among different diagnosis groups (Figure 3). *Post hoc* analysis was performed using the Dunn test (Table 3).

**TABLE 3:** Mutual comparison of individual diagnoses (Dunn test) for S NfL, CSF NfL, CSF Aβ_42_, CSF tTau, and CSF pTau; and significant differences between subgroups (*p* < 0.05).

Analyte	Diagnostic groups					
S NfL	Factor	1	2	3	4	5
	(*n*)	32	29	22	10	14
	Median	21.8	25.9	16.6	32.3	9.6
	Different from subgroups	(5)	(3) (5)	(2) (4)	(3) (5)	(1) (2) (4)
CSF NfL	Factor	1	2	3	4	5
	(*n*)	15	24	13	8	16
	Median	1,020	1,583	910	2,009	563
	Different from subgroups		(5)		(5)	(2) (4)
CSF tTau	Factor	1	2	3	4	5
	(*n*)	18	22	14	8	14
	Median	730	369	241	338	287
	Different from subgroups	(3) (5)		(1)		(1)
CSF pTau	Factor	1	2	3	4	5
	(*n*)	17	23	14	8	14
	Median	158	37	30	42	36
	Different from subgroups	(2) (3) (4) (5)	(1)	(1)	(1)	(1)
CSF Aβ_42_	Factor	1	2	3	4	5
	(*n*)	18	23	14	7	14
	Median	590	1,090	1,385	1,286	1,586
	Different from subgroups	(2) (3) (4) (5)	(1)	(1)	(1)	(1)

CSF NfL, cerebrospinal fluid neurofilament light chain; S NfL, serum neurofilament light chain; CSF Aβ_42_, cerebrospinal fluid β-amyloid peptide_42_; CSF tTau, cerebrospinal fluid total tau protein; CSF pTau, cerebrospinal fluid phosphorylated tau protein.

### 3.4 Assessment of interrater reliability among the investigated biomarkers

Since the compared methods had different RIs, we used Cohen’s kappa statistic to compare the assays based on clinical interpretation (Table 4). The highest kappa coefficients (indicating moderate conformity between the diagnostic kits) were found between the concentrations of S NfL and CSF NfL (κ = 0.480), and between CSF NfL and CSF tTau (κ = 0.351). The positive value was set at >10.5 ng.L^–1^ for the S NfL concentration, at >900 ng.L^–1^ for CSF NfL (value indicating axonal damage) (Arrambide et al., 2016), and at >452 ng.L^–1^ for CSF tTau (Bartoš et al., 2019).

**TABLE 4:** Assay conformity based on the kappa statistic.

	Kappa statistics conformity (%)	95% CI	SE
S NfL vs. CSF NfL	0.480	0.288 to 0.671	0.098
S NfL vs. CSF tTau	0.116	−0.005 to 0.237	0.062
S NfL vs. CSF pTau	0.171	0.051 to 0.290	0.061
S NfL vs. CSF Aβ_42_	0.080	0.021 to 0.140	0.030
CSF NfL vs. CSF tTau	0.351	0.177 to 0.525	0.089
CSF NfL vs. CSF pTau	0.144	−0.043 to 0.331	0.095
CSF NfL vs. CSF Aβ_42_	0.020	−0.123 to 0.162	0.073

Positive values were as follows: CSF NfL >900 ng.L^–1^ (Arrambide et al., 2016); S NfL >10.5 ng.L^–1^; CSF tTau >452 ng.L^–1^ (Bartoš et al., 2019); CSF pTau >46 ng.L^–1^ for 18–44 years and >66 ng.L^–1^ for 45–77 years (Bartoš et al., 2019); CSF Aβ_42_ <550 ng.L^–1^ (Bartoš et al., 2019). CSF NfL, cerebrospinal fluid neurofilament light chain; S NfL, serum neurofilament light chain; CSF Aβ_42_, cerebrospinal fluid β-amyloid peptide_42_; CSF tTau, cerebrospinal fluid total tau protein; CSF pTau, cerebrospinal fluid phosphorylated tau protein.

### 3.5 Stepwise multinomial linear regression analysis

We performed stepwise multinomial linear regression analysis to assess the effects of biochemical markers on MMSE values, and identified statistically significant effects of S NfL, CSF Aβ_42_, and CSF pTau (Table 5). The multiple *R*-squared value was 0.390, the adjusted *R*-squared was 0.349, and the *F* statistic was 9.594 at 3 and 42 df (*p* < 0.0001). These results indicated that 39% of the MMSE values can be explained by these three parameters, which each make different contributions to the final MMSE value.

**TABLE 5:** Final stepwise multinomial linear regression analysis.

Coefficients	Estimate	SE	*t*-Value	*p* (>| *t*|)
Intercept	24.741	2.163	11.440	6.52e−15***
CSF Aβ_42_	0.003	0.001	2.481	0.017*
S NfL	−0.125	0.047	−2.651	0.011*
CSF pTau	−0.018	0.008	−2.180	0.035*

MMSE = 24.741 + 0.003 × CSF Aβ_42_ − 0.125 × S NfL − 0.018 × CSF pTau. Multiple *R*-squared: 0.390. S NfL, serum neurofilament light chain; CSF Aβ_42_, cerebrospinal fluid β-amyloid peptide_42_; CSF tTau, cerebrospinal fluid total tau protein. Asterisk indicates the level of statistical significance.

## 4 Discussion

In this study, we tested S NfL as a marker of neuronal damage. We estimated the physiological levels of S NfL, and identify a cut-off value for assessing the degree of neuronal impairment, using a highly sensitive ELISA method. In healthy controls, S NfL was found to be dependent on age. Similar results have also been published using the SIMOA method (Hviid et al., 2020; Harp et al., 2022). Notably, we did not find age dependence of S NfL in groups of patients with varying degrees of cognitive deficit. The patients included in our study were older than the healthy controls; therefore, the age range was narrower among patients than in the healthy population. Importantly, some of the included diseases are often more severe in younger patients, such that the neuronal damage could be greater. These explanations are supported by our more detailed analysis of the age dependence S NfL in individual diagnostic groups, which revealed statistically significant age dependency in all subgroups, except those with non-Alzheimer dementia (Group 2). That subgroup included multiple diseases that affect younger people and progress more rapidly than AD (e.g., FTLD and CJD), which could have affected the results (Buganza et al., 2009; Mohandas and Rajmohan, 2009). We must also consider the impacts of other factors that affect S NfL levels (e.g., body mass index, renal function, blood volume, high-density lipoprotein, etc.) and are associated with increased risk of vascular disease (Koini et al., 2021), given that Group 2 also included several patients with vascular dementia classified as non-Alzheimer dementia (Román, 2005).

We also evaluated the correlation of S NfL as a biomarker of neuronal injury with other biomarkers in CSF in patients with various diseases that presented as dementia, movement disorder, or combination of both. Serum and CSF concentrations of NfL showed a strong correlation in all groups except AD (Group 1). We assume this may have been due to the small size of this group, and the low absolute concentrations of NfL in the examined tissues. Moreover, this group included a few patients with AD in the predementia state, who had lower NfL concentrations compared to patients with dementia, which could also have affected our results. The negative correlation between CSF NfL and MMSE score has previously been published (Das et al., 2022), and investigations of transgenic mice with familiar AD suggest that plasmatic NfL does not differ between presymptomatic animals and control mice (Loeffler et al., 2020). The uncertainty regarding plasmatic NfL levels (in contrast with CSF NfL) in preclinical AD has been mentioned in the literature (Andersson et al., 2020). Apart from this exception, our findings confirmed that the detection of serum NfL (without requiring CSF NfL examination) was sufficient in these diseases, in accordance with other recent studies (Mollenhauer et al., 2020; Delaby et al., 2022). In the AD group (Group 1), we found no significant correlation between S NfL and any other examined biomarker. This was quite surprising, especially in the case of tTau in CSF, because both of these biomarkers reflect neuronal damage (Holper et al., 2022). Dhiman et al. (2020) reported that they did not find an association between Aβ and NfL, but that tTau and pTau were each correlated with NfL. In our present study, we found a significant correlation between NfL and tau, only in the healthy population (Khalil et al., 2018). According to these results and the above-mentioned literature, it seems that S NfL is not suitable as early biomarker of AD alone. It is possible that repeated examinations and studies of the dynamics of S NfL will be more successful (Preische et al., 2019). It is important to realize that we studied serum biomarkers in different stages of the disease, from preclinical phase to severe dementia. We tried to take into account the disease duration from first sign to sample investigation. However, in this specific disease it is nearly impossible to gain precise time frame and family members are not able to determine even a year of symptomatic beginning. In the study of Giacomucci et al. (2022) there has been proved some dependence between NfL and Aβ, but there had been used SIMOA method. We found a significant correlation between S NfL and CSF Aβ_42_ only in the group with PD (Group 3). One possible explanation could be the absence of dementia in this group, and consequently higher concentrations of Aβ_42_ in the CSF of these patients (Siderowf et al., 2010; Irwin et al., 2013). In the groups with AD (Group 1) and combination disease (Group 4), there were negative regressions that reflect lower concentrations of Aβ_42_, while the NfL levels were increased.

As expected based on the high correlation between serum and CSF concentrations of NfL, we found similar patterns of correlation between CSF NfL and other biomarkers of neurodegenerative diseases. There was a significant correlation between CSF NfL and CSF tTau in the AD group (Group 1). We also found significant correlations between CSF NfL and CSF αS (in contrast with S NfL) in groups with synucleinopathies. Similar results were found in the studies of PD by Oosterveld et al. (2020), and of MSA by Tokutake et al. (2022).

We also examined the correlations of other biomarkers among themselves, independent of NfL. CSF Aβ_42_ and CSF tTau were correlated in the group with PD (Group 3) and controls (Group 5). This correlation has been previously shown among PD patients (Zhang et al., 2013). We also found strong correlations between CSF Aβ_40_ and CSF pTau in the general population of all patients (while the results were non-significant in individual groups), and between CSF Aβ_40_ and CSF tTau in the AD group (Group 1). The correlation between Aβ_40_ and CSF pTau among AD patients (and slightly in controls) was previously published by Lehmann et al. (2020). We obtained interesting results in our examination of CSF Aβ_40_ and CSF αS, with significant correlations in practically all groups. This correlation was previously reported in synucleinopathies (Swirski et al., 2014). Surprisingly, we also found correlations between CSF tTau and CSF αS in the groups with AD (Group 1), non-AD dementia (Group 2), and PD (Group 3), and among controls (Group 5). Synucleinopathies are characterized by decreased concentrations of αS in CSF (Mollenhauer, 2014), and we expected to find negative regression between markers of neuronal damage and accumulation of αS, as in the case of CSF NfL and CSF αS. Despite this assumption, the literature includes evidence of a correlation between CSF αS and tTau in early PD, where tTau and pTau were lower than in control patients (Kang et al., 2016).

Our study also included analysis of the dependence of S NfL concentration in different diagnostic groups (Table 3). Our findings enable differentiation of the healthy population (Group 3) from people with dementia (Groups 1, 2, and 4). It seems that the key element is cognitive performance, because S NfL concentrations could differentiate even PD (Group 3) and the combination of dementia and movement disorder (Group 4). A previous study also described this role of NfL (in CSF) in the distinction between parkinsonian syndromes and PD and healthy controls (Canaslan et al., 2021). Even serum NfL dynamics can predict cognitive decline in PD patients (Ma et al., 2021).

Analogically, we found similar results when we tested the NfL concentrations in CSF. However, CSF NfL did not allow us to distinguish the group with AD. A possible reason could be that this group included a few patients without dementia, and the concentrations of NfL in CSF are about 50 times higher than in serum. Notably, the determination is much more precise in this case.

The rest of the studied analytes are common biomarkers used for AD diagnostics: tTau, pTau, and Aβ_42_. Our results supported the general idea that tTau and pTau concentrations were high and Aβ_42_ concentration was decreased in CSF in AD (Jack et al., 2018). Each of these biomarkers could differentiate AD from other groups by itself.

Finally, the results of our stepwise multinomial linear regression analyses proved that S NfL, CSF Aβ_42_, and CSF pTau were related to MMSE scores.

Our study has several limitations. The highest limitation was the unavailability of a larger number of analyzed samples, and the heterogeneity of the groups with regards to the etiology of neuronal damage. In addition, for most of the neurodegenerative diseases, the definitive diagnosis can only be established post mortem.

## 5 Conclusion

In this study, we estimated the physiological levels of S NfL using a highly sensitive ELISA method. The S NfL ELISA assay has high sensitivity and specificity for assessing the neuronal damage. The results implied that measurement of the biomarkers CSF NfL and CSF tTau of patients with cognitive decline could be replaced by the less-invasive determination of S NfL. This could be useful in future therapeutic trials to monitor the disease course. S NfL levels reflected the severity of cognitive deficits assessed by MMSE. However, NfL is not specific to AD and according to our results it does not appear to be a suitable biomarker for early diagnosis of AD.

## Data availability statement

The raw data supporting the conclusions of this article will be made available by the authors, without undue reservation.

## Ethics statement

The studies involving humans were approved by the Ethics Committee of the Ostrava University Hospital, as a part of the project “Laboratory biomarkers of neurodegenerative diseases” (reference number 340/2021). The studies were conducted in accordance with the local legislation and institutional requirements. The participants provided their written informed consent to participate in this study.

## Author contributions

PK: Conceptualization, Data curation, Formal analysis, Funding acquisition, Investigation, Methodology, Project administration, Resources, Supervision, Validation, Writing – original draft, Writing – review & editing. RN: Conceptualization, Data curation, Formal analysis, Funding acquisition, Investigation, Methodology, Resources, Writing – original draft, Writing – review & editing. PB: Conceptualization, Funding acquisition, Investigation, Methodology, Resources, Validation, Writing – review & editing. KL: Formal analysis, Methodology, Writing – review & editing. MB: Resources, Supervision, Validation, Writing – review & editing. DS: Resources, Supervision, Validation, Writing – review & editing.
